# A Novel Membrane Protein-Specific Serine/Threonine Kinase: Tissue Distribution and Role in Sperm Maturation

**DOI:** 10.5402/2012/789105

**Published:** 2012-03-01

**Authors:** Debjani Nath, Arpita Bhoumik, Sujoy Das, Debdas Bhattacharyya, Sandhya R. Dungdung, Gopal C. Majumder

**Affiliations:** ^1^Cell Biology & Physiology Division, Indian Institute of Chemical Biology, Jadavpur, Kolkata 700032, India; ^2^Department of Zoology, University of Kalyani, Kalyani 743215, India; ^3^Cryobiology Department, Centre for Rural and Cryogenic Technologies, Jadavpur University, Kolkata 700032, India

## Abstract

Our recent studies have described for the first time the purification of an ectoprotein kinase to apparent homogeneity using caprine sperm as the model. Purified ectokinase (CIK) is a novel membrane protein-specific kinase that phosphorylates serine and threonine residues of ectophosphoproteins. This study, using ELISA based on ecto-CIK antibody demonstrates that ecto-CIK level is remarkably higher in the sperm membrane than in the cytosol. The epididymal sperm maturational event as well as sperm vertical velocity is associated with a significant increase in the ecto-CIK level. Ecto-CIK, the membrane protein-specific kinase, is also present in all the tissues tested and is predominantly localized in the cell membrane. Ubiquitous localization of the novel kinase on the mammalian cell membrane suggests that the kinase may play pivotal role in gamete as well as somatic cell regulation by modulating membrane biology through serine/threonine phosphorylation of specific membrane proteins located in the ectodomains.

## 1. Introduction

 Testicular spermatozoa are immotile and undergo maturation during transit through the caput, corpus, and cauda parts of the epididymis where they acquire forward motility and fertility potential. Studies from our laboratory have provided several lines of evidence for the occurrence of a cyclic AMP-independent protein kinase (ecto-CIK) on the external surface of caprine (Capra indicus) cauda epididymal mature spermatozoa that causes phosphorylation of the membrane-bound phosphoproteins [1–3]. Our recent studies using caprine sperm as the model have described for the first time the purification to apparent homogeneity of an ectoprotein kinase [4] as well as its major phosphoprotein substrate: MPS [5] located on the sperm external surface. Ecto-CIK is a 115 kDa protein made up of two subunits: 63 and 55 kDa. It is a strongly basic protein. It is also strongly immunogenic. The ectokinase has nearly 30 times greater affinity for MPS as compared to casein—the most potent exogenous protein substrate. Thereby, it demonstrates that CIK is a unique membrane protein-specific kinase, which specializes for phosphorylating the serine and threonine residues of the sperm outer cell-surface phosphoproteins [5]. MPS is a 100 kDa phosphoprotein. Both CIK and MPS are localized primarily in the acrosomal cap area of the external surface of the mature sperm head as demonstrated by indirect immunofluorescence studies [4, 6]. We have demonstrated that ecto-CIK through its substrate protein (MPS) plays a vital role in the regulation of sperm forward progression and velocity [4, 5, 7]. More recent studies from our laboratory demonstrated that the sperm external surface CIK and MPS are essential for membrane fusion component of acrosome reaction [6].

 The present study using the purified antibody of CIK in the ELISA technique reports tissue specificity of the ectokinase. It also demonstrates the epididymal maturational profile of caprine sperm during its journey through the epididymis.

## 2. Materials and Method

### 2.1. Reagents

 The following reagents were obtained from Sigma Chemical Company (St. Louis, MO): Polyethylene glycol (average molecular weight 20 kDa), ^32^P-labeled ATP, ethylenediaminetetra-acetic acid (EDTA), phenyl methyl sulphonyl fluoride (PMSF), casein-sepharose-4B, PBE-118, triethyl amine-HCl, *β*-mercapto ethanol, glycerol, Triton X-100, DEAE cellulose, HRP-conjugated anti-rabbit IgG, sodium chloride, potassium chloride, potassium phosphate, penicillin, complete Freund's adjuvant, incomplete Freund's adjuvant, and ammonium sulphate. Phosphate buffer saline (PBS), orthophenylenediamine (OPD), Citrate phosphate Buffer, H_2_O_2_, H_2_SO_4_, BSA, fetuin, casein, ovalbumin, Calcium chloride, Magnesium sulphate, Glucose, Sodium bicarbonate, Sucrose.

### 2.2. Isolation of Caprine Epididymal Spermatozoa

 Goat epididymal spermatozoa were isolated within 2 hours of slaughter [8]. To obtain the mature sperm, cauda epididymis was cut to several pieces and suspended in a modified Ringer's solution (RPS medium: 119 mM NaCl, 5 mM KCl, 1.2 mM MgSO_4_, 10 mM glucose, 16.3 mM potassium phosphate, and 50 U penicillin mL^−1^, pH 6.9) with gentle stirring. Maturing spermatozoa were derived from caput and corpus epididymis. The spermatozoa were then filtered through 4 to 5 layers of cheese cloth and sedimented by centrifugation at 500 g for 5 minutes and then washed in RPS medium.

### 2.3. Isolation of Goat Sperm Cytosol and Plasma Membrane

Sperm cells after washing in RPS medium were sonicated and then centrifuged at 17,000 g for 15 minutes at 4°C. The supernatant was designated as cytosol. Pellet was used as source of plasma membrane. Highly purified plasma membranes were isolated from the mature and maturing sperm by an aqueous two-phase polymer method [9]. The membrane preparation was finally dispersed in “buffer A”: 25 mM potassium phosphate buffer, pH 7.0, containing 1 mM PMSF, 2 mM *β*-mercapto ethanol, 1 mM EDTA, and 30% (v/v) glycerol and was stored at −20°C. The protein content of the plasma membrane was estimated using BSA standard [10].

### 2.4. Purification of Membrane-Bound Ecto-CIK

The ecto-CIK was purified to apparent homogeneity from the plasma membrane of mature cauda epididymal spermatozoa [4]. Isolated plasma membrane was solubilized using 1% Triton X-100 and kept in ice for 1 hour and centrifuged at 27000 g for 90 minutes at 4°C. The resulting supernatant was loaded on DEAE-Cellulose column. The activity peak, eluted in unretained fraction, and chromatographed on casein-Sepharose 4B affinity column. The activity peak was eluted with 0.2 M NaCl, loaded on PBE-118 column equilibrated with 0.025 M triethyl amine-HCl, pH 11.0. The activity peak was eluted depending on its PI. The active fractions were concentrated and passed through casein Sephsrose-4B column again to remove the eluent buffer. The active fraction was isolated as before, dialysed and concentrated, and kept in buffer A with 50% glycerol at −20°C until used.

### 2.5. Production of Antibody

 Antiserum against the purified CIK was raised in rabbit by 4 successive injections at the 1st, 7th, 15^th^, and 21st days as described earlier [4]. First injection was given subcutaneously using 500 *μ*g of CIK in complete Freund's adjuvant. Second and third injections comprised 200 *μ*g of CIK in incomplete Freund's adjuvant. Fourth injection contained 400 *μ*g of CIK in incomplete Freund's adjuvant. Blood was collected from the ear vein on the 27th day of inoculation, and serum was prepared and stored at −70°C. Preimmune blood serum was collected from the same animal before starting the inoculation programme [11]. The immunoglobulin of the immune serum was precipitated twice with 50% ammonium sulfate. The final precipitate was dissolved in 0.25 M PBS (pH 8.0) and dialyzed overnight against the same buffer. The immunoglobulin fraction obtained after the ammonium sulfate fractionation was subjected to DEAE-cellulose chromatography. Unbound protein peak containing IgG was collected with.01 M phosphate buffer, pH 7.0, and stored at −20°C.

### 2.6. Preparation of Cytosol and Cell Membrane Fractions of Different Tissues

 Cytosol and cell membranes were obtained from goat liver, lung, kidney, heart, bone marrow, testis, muscle, and spleen. All tissues except epididymis were thoroughly washed in RPS medium (119 mM NaCl, 5 mM Kcl, 1.2 mM MgSO_4_, 10 mM glucose, 16.3 mM potassium phosphate buffer, pH 6.9, penicillin, 50 units/mL) to remove blood contamination. In case of epididymis, the tissues were first minced and washed in RPS medium. The buffer containing sperm and EP were removed by decantation. This procedure was repeated several times until it became free of luminal fluid and sperm as examined under microscope. Washed tissues were then gently homogenized in a polytron homogenizer in 2 mL PBS, pH 7.0, and the homogenates were then centrifuged at 200 g for 10 minutes at 4°C. The resulting supernatants were then centrifuged at 1,00,000 g for 60 minutes at 4°C to obtain cytosol.

Purified plasma membranes from liver, lung, heart, muscle, spleen, bone marrow, kidney, and testis were isolated by sucrose gradient method according to Emmelot et al. [12].

### 2.7. ELISA of CIK

 The protein content of the tissue samples was estimated according to Bradford's method [13] using bovine serum albumin as standard. 50 mL of PBS (10 mM sodium phosphate, pH 7.5, containing 0.9% NaCl) was added for blank and variable amounts of different tissue extracts (0.5–100) were added to microtitre plate so that the experiment follows linear range of assay and incubated overnight at 4°C. After washing with PBS, the wells were blocked with PBS containing 3% BSA and incubated at 37°C for 1 hour. Then the 1st antibody (CIK antibody) in PBS containing 1% BSA was added. Incubation and washing was done as before followed by the addition of HRP-conjugated goat anti-rabbit IgG (2nd antibody at a dilution of 1 : 1000 in PBS containing 1% BSA). Then the plate was incubated at 37°C for 1 hour. Finally colour development was done by using 3 mM orthophenylenediamine (OPD) in 24 mM citric acid-50 mM disodium hydrogen phosphate containing 0.04% H_2_O_2_ (pH 5.1–5.4) in PBS [14]. Development of colour was stopped after 30 minutes with 4(N) H_2_SO_4_, and absorbance was measured at 492 nm by ELISA reader. Specific activities of the membrane-specific kinase in the tissue fractions were expressed as OD/*μ*g proteins.

## 3. Results

 For evaluating immunological protein specificity of antibody of CIK (raised in rabbit), several commercially available proteins like BSA, fetuin, casein, and ovalbumin were tested by the conventional immunoblot technique. Only CIK gave color in the immunoblot whereas other proteins did not show any appreciable color (data not shown), indicating thereby that antibody has high immunological specificity for CIK. This antibody has been used in this investigation with specific reference to sperm maturational profile and tissue distribution.

### 3.1. Distribution of CIK in Sperm Cytosol and Plasma Membrane

 Previous studies from our laboratory using caprine (*Capra indicus*) sperm as the model showed that CIK is localized on the sperm external surface and it causes phosphorylation of the outer cell-surface phosphoproteins [1–4, 15].  Table 1 shows the distribution of CIK in the mature cauda sperm cytosolic and plasma membrane compartments as measured by the ELISA technique.

### 3.2. Sperm Maturational Profile of CIK

**Figure 1:**
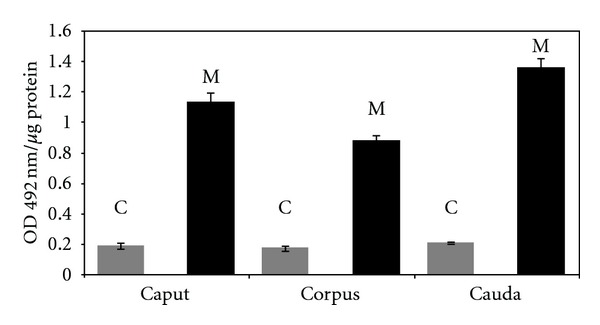
Immunoassay of CIK in maturing caprine sperm (i.e., caput, corpus, and cauda sperm) cytosol and plasma membrane: sperm cytosol and plasma membrane fractions were prepared as described in Section 2. Sperm plasma membranes were isolated by two-phase polymer method. Distribution of CIK in the sperm fractions was determined by ELISA under standard assay condition. The data shown are Mean ± s.e.m. of 5 experiments. “C” denotes cytosol and “M” denotes membrane.

Figure 1 shows the profile of ecto-CIK as the caprine sperm undergoes maturation in epididymis. The levels of the protein kinase were significantly (*P* < 0.001) higher in the mature sperm than in the maturing male gametes. The maturational pattern of the ecto-kinase is essentially similar in both cytosol and cell membrane. It is of interest to note that during the initial phase of the epididymal transit (caput to corpus) there is significant (*P* < 0.001) fall in the levels of the protein kinase in cytosol and membrane (approx. 20%), followed by a sharp rise in the level of the CIK (approx. 50%) in both the cytosol and the membrane of mature cauda sperm. The membrane-bound CIK was nearly 5-fold higher than the cytosolic kinase in all the maturing sperm cells.

### 3.3. CIK Level in “Vertically” Motile Sperm

**Figure 2:**
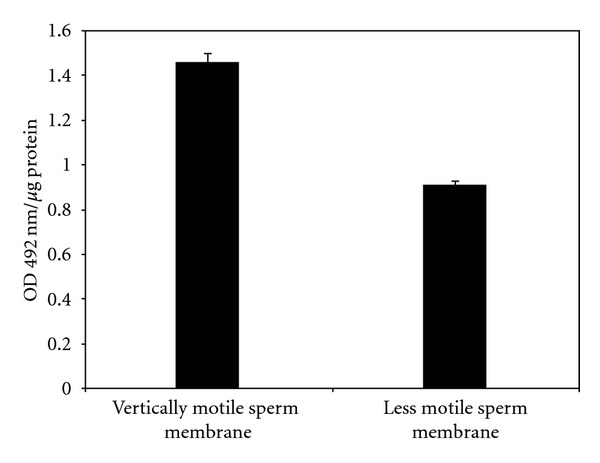
Immunoassay of CIK in vertically motile sperm and weakly motile/nonmotile sperm plasma membrane: spermatozoa from the cauda epididymis were suspended in modified Ringer solution containing 2% Ficoll and were placed at the bottom of a beaker containing modified Ringer's solution. Vertically motile sperms were then collected by swim up technique. Sperm plasma membranes were prepared as described in Section 2. Distribution of CIK was determined by ELISA under standard assay condition. The data shown are Mean ± s.e.m. of 5 experiments.

 Vigorously motile sperm cells have been separated from the weakly motile/nonmotile spermatozoa by the swim-up technique, and both these groups of cell, were assessed for their CIK contents (Figure 2). The specific activity of the membrane-bound ecto-CIK is significantly (*P* < 0.001) higher (nearly 2-fold) in the “vertically” motile sperm than the weakly motile/nonmotile spermatozoa.

### 3.4. Distribution of CIK in Cytosolic and Plasma Membrane Fractions of Different Tissues

**Figure 3:**
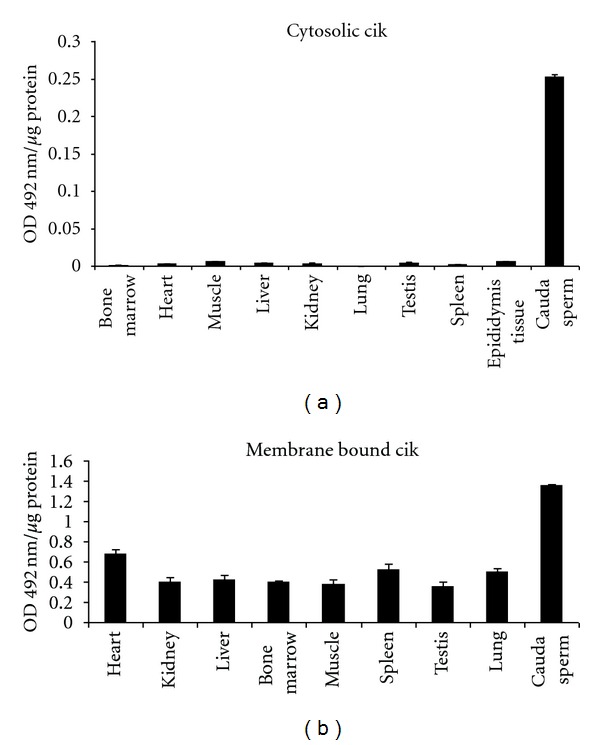
(a) Immunoassay of CIK in various tissue cytosol: extracts were prepared from samples of goat liver, lung, kidney, heart, bone marrow, and testis as described in Section 2. Distribution of CIK was determined by ELISA under standard assay condition. The data shown are Mean ± s.e.m. of 5 experiments. (b) Immunoassay of CIK in various tissue plasma membranes: plasma membranes from goat liver, lung, kidney, heart, lung, muscle bone marrow, and testis were prepared according to Section 2. Distribution of CIK was determined by ELISA under standard assay condition. The data shown are Mean ± s.e.m. of 5 experiments.

 The levels of CIK in the cytosolic fractions of different caprine tissues and mature cauda spermatozoa were investigated by ELISA. Of all the tissue extracts tested sperm cytosol showed the highest level of ecto-CIK. The specific activity of sperm cytosolic CIK was nearly 40-fold higher than epididymis and approximately 100-fold higher than most of the other tissues. Epididymis has the 2nd highest level of CIK whereas all other tested tissues such as the liver, spleen, heart, kidney, lung, muscle, testis and bone marrow have low level of the kinase (Figure 3(a)). As epididymis is the site of maturation of spermatozoa, sperm cell extracts and sperm plasma membrane were then analysed for CIK level.

As the kinase is primarily localised on the sperm cell membrane (Table 1, Figure 1), it is worthwhile to investigate the specific activity of the kinase in the purified plasma membranes derived from different tissues (Figure 3(b)). The results clearly reveal that like the membrane of the male gamete, other cell membranes are also rich in the ecto-CIK. Of the eight tissues tested, heart membrane elicited the highest specific activity of the CIK. The level of the kinase in cauda sperm membrane is nearly twofold higher than that of the heart. In general, the level of the CIK in cell membrane is nearly 200-fold higher than in the cytosolic fractions.

### 3.5. CIK Levels at Different Segments of Epididymis

**Figure 4:**
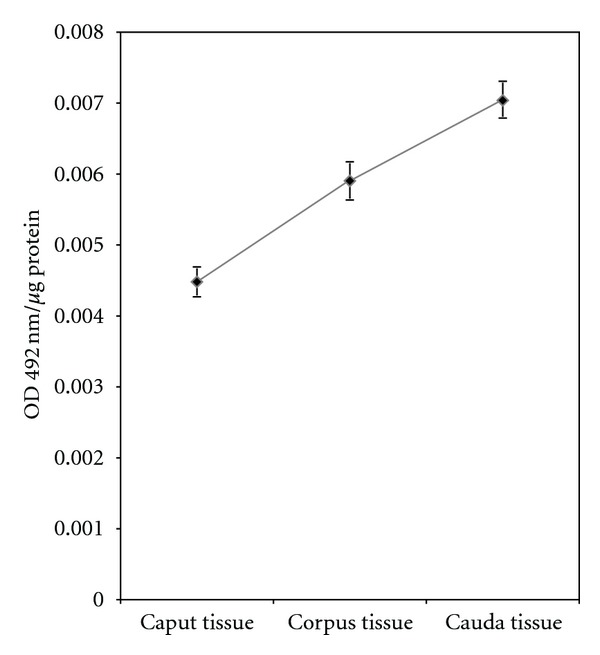
Immunoassay of CIK in different segments of epididymis: extracts were prepared from different segments of epididymis, that is, caput, corpus, and cauda as described in Section 2. Distribution of CIK was determined by ELISA under standard assay condition. The data shown are Mean ± s.e.m. of 5 experiments.

 Immature testicular spermatozoa acquire their forward progression characteristics as they pass through the caput, corpus, and cauda parts of epididymis. As mentioned before, among the traditional organs, epididymis has the highest level of the CIK. It is thus of interest to analyze the CIK content in different parts of this organ through which sperm is in transit during maturation.  Figure 4 indicates that the level of the kinase increases progressively in the epididymal segments from the caput to cauda.

## 4. Discussion

 Many investigators have provided several lines of evidence to support the occurrence of multiple types of protein kinases in a variety of mammalian cells. Cyclic AMP-dependent and independent protein kinases are the two major classes of the kinase that have been demonstrated on the external cell surface [1, 16–23]. The cyclic AMP-independent kinase group are the most extensively studied enzymes from the standpoint of phosphorylation of endogenous membrane proteins [3, 23–27] and regulation of cellular functions [26, 28, 29]. A major limitation in earlier investigations was that the precise biochemical identity of ectokinases and their specific endogenous ectoprotein substrates were largely unknown as the kinases as well as their physiological protein substrates were not purified. Our recent studies have described for the first time the purification of an ectoprotein kinase (ecto-CIK) [4] from caprine sperm plasma membrane to apparent homogeneity. CIK is a unique membrane protein-specific serine/threonine kinase. This study, using ELISA based on the antibody of the purified sperm ecto-CIK, reports the distribution pattern of the CIK in sperm cytosol and the membrane as the sperm acquires forward progression during epididymal maturation. This investigation also reports for the first time tissue specificity of this novel membrane protein-specific kinase.

The level of ecto-CIK is remarkably higher in the membrane than in the cytosol (Table 1) showing that the kinase following its synthesis in the cytosol is translocated substantially to the external cell surface. The specific activity of the ectoprotein kinase in cell membrane was significantly higher in the mature cauda sperm than in the immature caput spermatozoa (Figure 1) thereby revealing that epididymal initiation of sperm forward motility is associated with the ecto-CIK. Cytosolic level of the ecto-CIK also increases significantly during sperm maturation. During the early maturational phase (transit from the caput to corpus), CIK content both in cytosol and cell membrane decreases significantly prior to a sharp coordinated rise in mature sperm. Relatively high level of CIK in epididymal cytosol as compared to other tissues (Figure 3(a)) suggests that the epididymis may serve as the source of CIK for supplying to the maturing sperm while in transit through this organ. Significant progressive enhancement of the specific activity of CIK in the epididymis (cytosol) from the caput to cauda parts (through which spermatozoa pass through during maturation) (Figure 4) is consistent with the view that the epididymis plays an important role in sperm maturational event by providing CIK.

Previous studies from our laboratory have shown the occurrence of ecto-CIK-coupled enzyme system made up of an ecto-CIK and its protein substrates and a PPase that causes phosphorylation and dephosphorylation of sperm external surface phosphoproteins [1, 4, 30, 31]. There was a marked decrease in the ecto-CIK activity of the intact spermatozoa as these cells undergo maturation in the epididymis. This observation is based on the assay of cell-bound CIK activity using endogenous ectophosphoproteins as protein substrate as well as saturating amount of phosvitin: a potential exogenous substrate of the kinase [32]. A similar phenomenon was also observed when CIK activity was estimated in isolated membranes of the maturing sperm [33]. Like the ecto-CIK, ecto-PPase activity also undergoes marked decrease during sperm maturation [34]. PPase interferes in the protein kinase assay as it dephosphorylates phosphoproteins. It is thus clear that the above-mentioned earlier reports on the maturation-dependent alteration of intact sperm and isolated membrane ectoprotein kinase activities and membrane protein phosphorylation [32, 33] are the resultant effects of the kinase and PPase activities. The above contention is likely to be applicable to all the reported studies in a variety of mammalian cells under altered physiological states (for reviews see [4]) as PPase has also been demonstrated in isolated membranes and on external cell surface [35–38]. Ecto-ATPase of the intact live cells and isolated cell membranes complicates further the situation because of its role in restricting the availability of ATP needed for protein phosphorylation. All these earlier investigations thus represent “apparent” rather “true” values of ecto- or membrane-bound protein kinase activities/membrane protein phosphorylation patterns under various physiological alterations. As elaborated below, availability of specific antibody against the above-mentioned cell surface antigens (protein kinase and its substrates, etc.) will be extremely useful to resolve the above-mentioned technical mess that has not been adequately addressed earlier.

Availability of specific CIK antibody will now be extremely useful to estimate the “actual” CIK content on intact cell and isolated membrane. As mentioned above, there was a marked sperm-maturation-dependent fall in the ecto-CIK activity of the live spermatozoa when the kinase was estimated using phosvitin as the exogenous protein substrate [2]. Under this assay condition availability of endogenous protein substrate on the cell surface may not be a significant variable, as there was saturating level of phosvitin: a potential exogenous substrate of the kinase. Epididymal maturation-dependent fall in CIK activity can thus be attributed to primarily remarkable decrease in the available CIK catalytic site on the cell surface. But the actual situation as revealed by the ELISA data (Figure 1) is quite different. During sperm maturation the ecto-CIK content in fact increases significantly. The biochemical basis of this apparent anomaly is not clear. As reported earlier, there is a remarkable topographical alteration of ecto-CIK during epididymal transit of the male gametes [4]. During the maturation event there is a profound lateral movement of ecto-CIK that gets finally “capped” primarily on the acrosomal tip of the mature cauda sperm head thereby reducing remarkably the surface exposure of CIK. This observed modulation of sperm surface topography of the kinase appears to be the major parameter restricting the availability of sperm surface CIK catalytic sites as the sperm maturation progresses in epididymis. Mechanism of this maturation-dependent restructuring of the ecto-CIK on the sperm external surface is not known.

Our earlier study showed that vigorously motile sperm has markedly higher ecto-CIK activity when assayed with exogenous protein substrate and intact sperm-bound endogenous phosphoprotein substrates of the cell surface [2] despite the finding that spermatozoa with vertical velocity also possess high level of ecto-PPase activity [39]. Using specific antibody of CIK, it has been shown that the ectokinase is required for the regulation of sperm velocity [4]. This observed correlation of ecto-CIK with sperm vertical velocity is apparently not consistent with the earlier observation that the intact sperm and isolated membrane ecto-CIK activity [32, 33] decreases markedly during epididymal motility initiation. Maturation-dependent increase of the ectokinase level (Figure 1) and the finding that the vertically motile sperm possesses significantly higher level of the membrane-bound CIK than the weakly motile/nonmotile spermatozoa (Figure 2) strengthen the view that CIK is associated with sperm forward motility right from its initiation in the epididymis. The present findings generated with the application of purified CIK antibody thus resolve an apparent inconsistency in the literature.

Like spermatozoa, the membrane protein-specific kinase, ecto-CIK, is present in all the tissues tested (Figure 3). It is present at a very low level in cytosolic fraction and is predominantly localized in the plasma membrane of all the cells. This study thus demonstrates for the first time the occurrence of the ecto-CIK in relatively high proportion not only in the sperm plasma membrane but also in other mammalian cells. Ubiquitous localization of the unique protein kinase in the mammalian cell membrane is consistent with the view that it may play vital role in cellular regulation by modulating the structure and function of biomembrane through phosphorylation of serine/threonine residue of specific proteins oriented to the external cell surface. Being a novel plasma membrane-specific serine/threonine protein kinase, CIK may cause phosphorylation of specific membrane proteins and thereby may modulate membrane biology of the mammalian cells. This study (Figures 1 and 2) along with the earlier ones has already established the importance of ecto-CIK in sperm motility [4, 6] and membrane fusion event of sperm acrosome reaction [6], which is essential for fertilization of oocytes. Like the male gametes, the physiological functions of the somatic cells may as well be regulated by this unique membrane-specific protein kinase. CIK and its specific substrates because of their localization in the ecto-domains of the cells may play vital role in the regulation of cell-cell interactions, recognitions, signal transduction, and so forth.

## Figures and Tables

**Table 1:** Distribution of CIK in the mature cauda sperm cytosolic and plasma membrane: sperm cells after washing in RPS medium were sonicated and then centrifuged in high-speed centrifuge. The resulting supernatant is sperm cytosol. Sperm plasma membranes were isolated by a two-phase polymer method. Distribution of CIK was determined by ELISA as described in Section 2. These data shown are for 5 experiments.

Sperm fractions	OD 492 nm/*μ*g protein ± SEM
Cytosol	0.253 ± 0.025
Plasma membrane	1.363 ± 0.02
